# Novel duck reovirus exhibits pathogenicity to specific pathogen-free chickens by the subcutaneous route

**DOI:** 10.1038/s41598-021-90979-w

**Published:** 2021-06-03

**Authors:** Kexiang Yu, Jinfeng Ti, Xiao Lu, Li Pan, Liping Liu, Yuehua Gao, Xiaozhen Guo, Feng Hu, Cunxia Liu, Xiuli Ma, Yufeng Li, Bing Huang, Minxun Song

**Affiliations:** 1 Institute of Poultry Science, Shandong Academy of Agricultural Sciences, No. 1 Jiaoxiao Road, Jinan City, 250023 Shan Dong Province China; 2 Shandong Provincial Key Laboratory of Immunity and Diagnosis of Poultry Diseases, Jinan City, Shan Dong Province China; 3 Shandong Vocational Animal Science and Veterinary College, Jinan City, Shan Dong Province China

**Keywords:** Microbiology, Virology, Viral pathogenesis

## Abstract

To study the pathogenicity of new duck reovirus (NDRV) to chickens, eighty 3-day-old SPF chickens were equally divided into two groups. The experimental group was inoculated with a NDRV challenge strain of 100 μL (10^–5.00^ ELD_50_/0.1 mL) by the subcutaneous (s.c.) route, and the control group was inoculated with 100 μL of sterile phosphate-buffered saline (PBS) by the same route. In the experimental group, chickens exhibited introflexion of claws, performing of splits, stunting syndrome, weight loss and death. Gross lesions such as enlargement and yellowish-white focal necroses were observed in the liver and spleen. Microscopic changes were typical including varying degrees of hepatocyte steatosis and necrosis, splenic lymphocyte necrosis, interstitial pneumonia. Viral loads were detected in lung, liver, heart, spleen, duodenum, burse and kidney. The liver and spleen viral loads remained a much higher level and maintained for a longer time, suggesting that these tissues might be the target organs. In summary, NDRV can cause systemic infections and death in chickens, which indicated that chickens may be infected by NDRV in poultry production.

## Introduction

Avian reovirus (ARV), belonging to Orthoreovirus, family Reoviridae, infects chickens, turkeys, ducks, geese and other birds^1,2^. The virus exists all over the world, and the current ARV mainly causes three different pathogenic types in China^3^. Chicken viral arthritis and short syndrome caused by chicken reovirus (CRV) mainly results in arthritis, tenosynovitis and retarded growth^4^. Muscovy ducks infected by Muscovy duck reovirus (MDRV) mainly manifested many yellowish-white or white necrotic foci in the liver, and sometimes bleeding points accompanied them. Therefore, the disease is commonly known as flower liver disease^5,6^. The spleen necrosis disease of ducks and geese caused by a new type of duck reovirus (NDRV) mainly presented as haemorrhage and necrosis in the liver and spleen^7–9^. The diseases caused by the three types of ARV manifested much greater differences either from the epidemiology, clinical symptoms and pathological changes or from the gene sequence and serology of the three pathogens^3^.

NDRV mainly causes the death of ducklings. No clinical symptoms have been observed in infected adult ducks. The disease originally appeared in ducks from some Chinese southern provinces, such as Fujian, Guangdong, Zhejiang and so on, in 2005^10,11^. Soon the disease spread throughout the main duck farms of China, and now has already became a common and frequently occurring disease. The onset age of the disease was generally 5–25 days old, especially 7–14 days, the morbidity was 5–35%, and the mortality was 2–20%. In general, the younger that the onset ages of the ducks were, the higher that the morbidity and mortality were^10,11^. The virus can cause spleen necrosis and pathological injury of the bursae, which can result in immunosuppression of the body and tends to cause secondary bacterial infections^12–14^. Thus, the disease is much more difficult to control and frequently resulted in a higher mortality rate of diseased ducks. The disease caused great economic losses for the Chinese duck industry.

NDRV can infect the Cherry Valley duck, Shelduck, Muscovy duck, mule duck, duck, goose and other waterfowl species^15^. It was reported that NDRV can infect chicken embryos and cause obvious lesions of the liver, spleen and bursae^16^. However, the pathogenicity of NDRV to chickens has not been reported. In this study, we explored the pathogenicity of the virus to chickens by inoculating NDRV in 3-day-old SPF chickens subcutaneously, which could lay the foundation for better preventing and controlling the disease.

## Results

### Clinical symptoms and body weight changes

**Figure 1:**
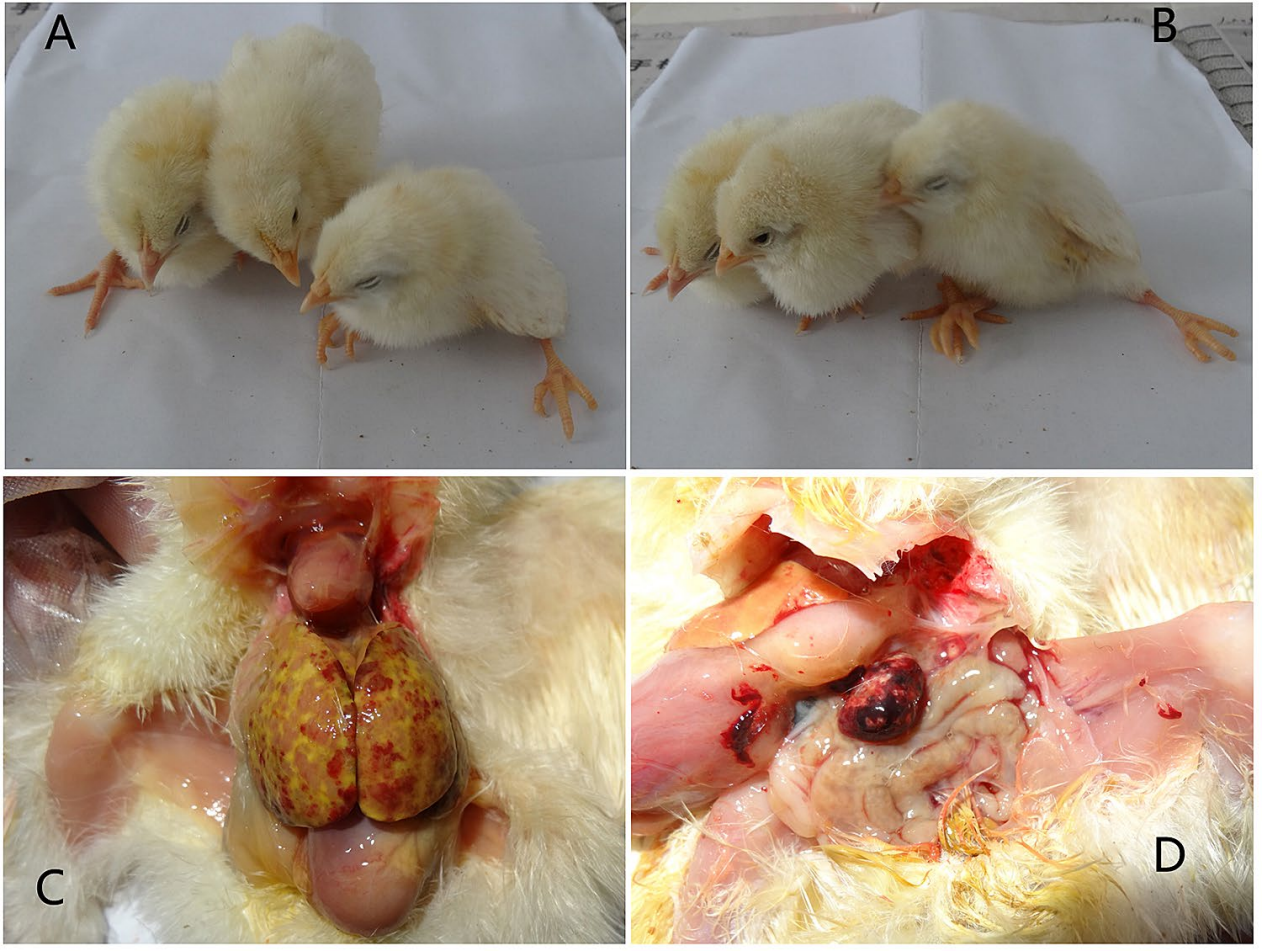
Clinical signs and gross lesions of chickens infected with NDRV by the s.c. route. (**A**) Chickens displayed depression, craws curling at 3 dpi. (**B**) Chickens displayed performing splits at 3 dpi. (**C**) Yellowish-white focal necrosis on the surface or in the parenchyma of liver. (**D**) Swelling, bleeding and yellowish-white focal necrosis on the surface or in the parenchyma of spleen.

**Figure 2:**
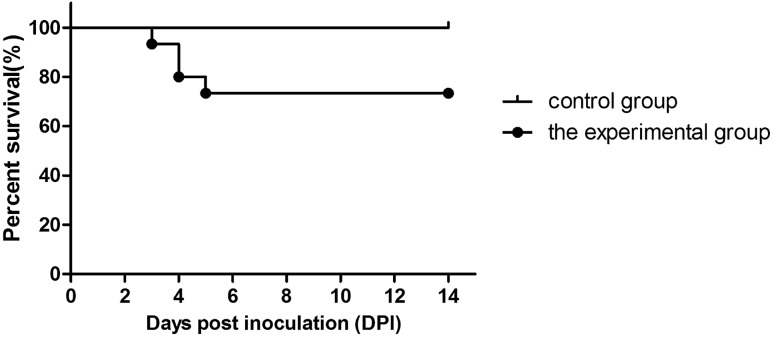
Survival rate of chickens infected with NDRV by the s.c. route. Five chickens died during 3 to 5 days after infection.

**Figure 3:**
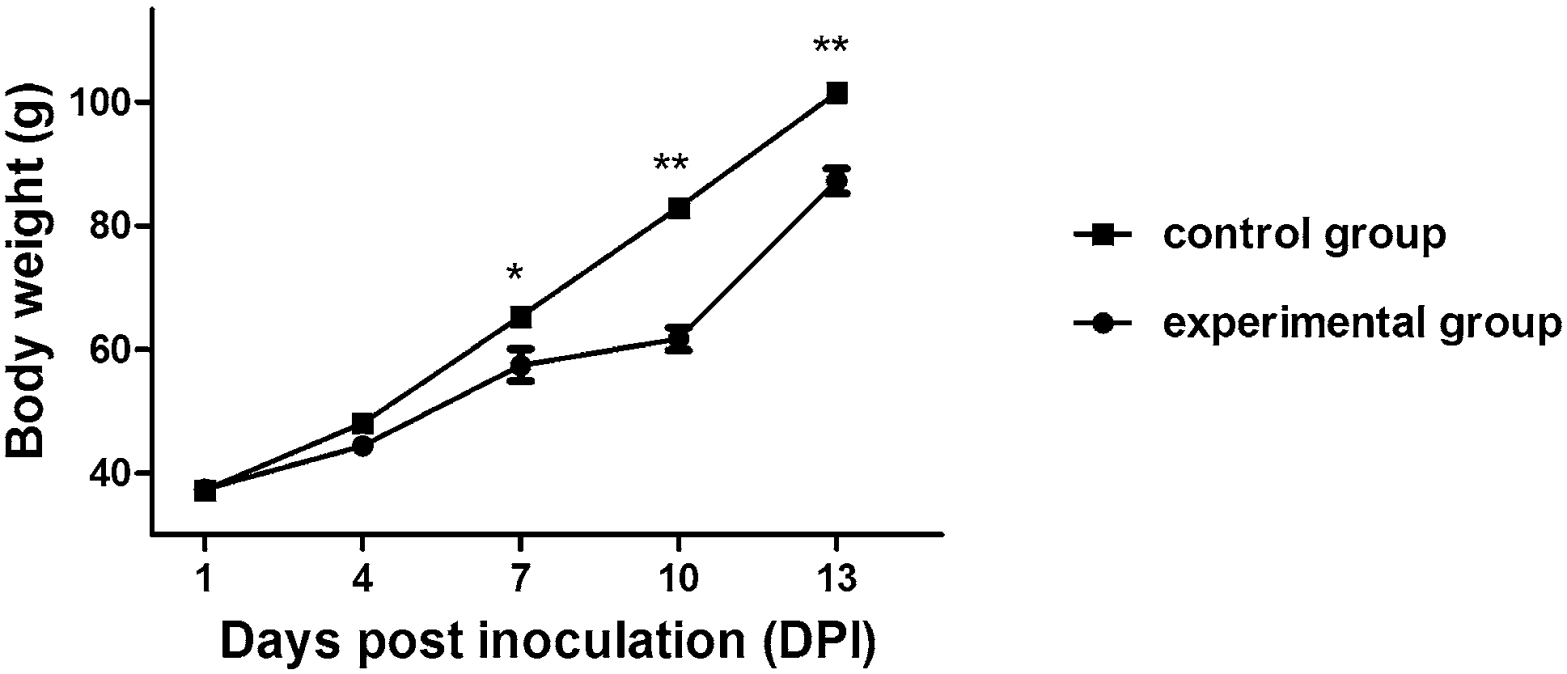
Body weight changes for chickens infected with NDRV by the s.c. route. The experimental group chickens were infected with 100 µL × 10^5.00^ ELD_50_/0.1 mL of NDRV. The control group chickens were infected with 100μL PBS (pH 7.0). Bars show means ± standard deviation (SD). The mean value was statistically significant, determined using by the two-tailed Student’s unpaired t-test (*P < 0.05 and **P < 0.01).

Forty 3-day-old SPF chickens in the experimental group were inoculated with 100 μL of allantoic fluid (10^5.00^ ELD_50_/0.1 mL) of NDRV by the s.c. route. The chickens in the experimental group began to exhibit depression, reluctant activities, introflexion of claws (Fig. 1A), and performing of splits (Fig. 1B) at 3 dpi. Five chickens died from 3 to 5 days after infection, and the other chickens presented with stunting syndrome (Fig. 2). Ten chickens were randomly selected from each group and weighed every four days for 14 days. The body weight of the experimental group was statistically significant lighter than that of the control group and there were significant differences between them. The mean body weight decreased to 7.73–25.64% compared to the control group (Fig. 3). From 10 dpi, the body weight gain started to recover, and drinking and eating began to return gradually in the experimental group. Chickens in the control group did not show any clinical signs.

### Gross lesions

At 3, 6, 9 dpi, five chickens from each group were euthanized by carbon dioxide. Gross lesions are summarized in Table 1. In detail, all infected chickens at 3 and 6 dpi, as well as 4/5 chickens at 9 dpi showed severe lesions in the liver and spleen. Hepatomegaly and brittleness were seen in the liver, and there were many yellowish-white focal necroses of variable size, on the surface or in the parenchyma of the liver (Fig. 1C). The spleen was swollen, showed haemorrhages, and multifocal yellowish-white necroses, up to 3 mm in diameter, present on the surface or in the parenchyma (Fig. 1D). No gross lesions were found in other organs of experimental group or in any organ of the control group.

**Table 1:** Gross lesions in chickens of two different groups at 3 dpi, 6 dpi and 9 dpi.

Organ	Experimental group			Control group		
	3 dpi	6 dpi	9 dpi	3 dpi	6 dpi	9 dpi
**Liver**						
Lesion rate	5/5^a^	5/5	4/5	0/5	0/5	0/5
Evaluation	+++^b^	+++	+++	–	–	–
**Spleen**						
Lesion rate	5/5	5/5	4/5	0/5	0/5	0/5
Evaluation	+++	+++	+++	–	–	–

^a^Number of chickens exhibiting gross lesions in the sacrificed chickens infected with NDRV.

^b^Severity of gross lessions: −, no gross lesions; +, mild gross lesions; ++, moderate gross lesions; +++, severe gross lesions.

### Microscopic lesions

**Figure 4:**
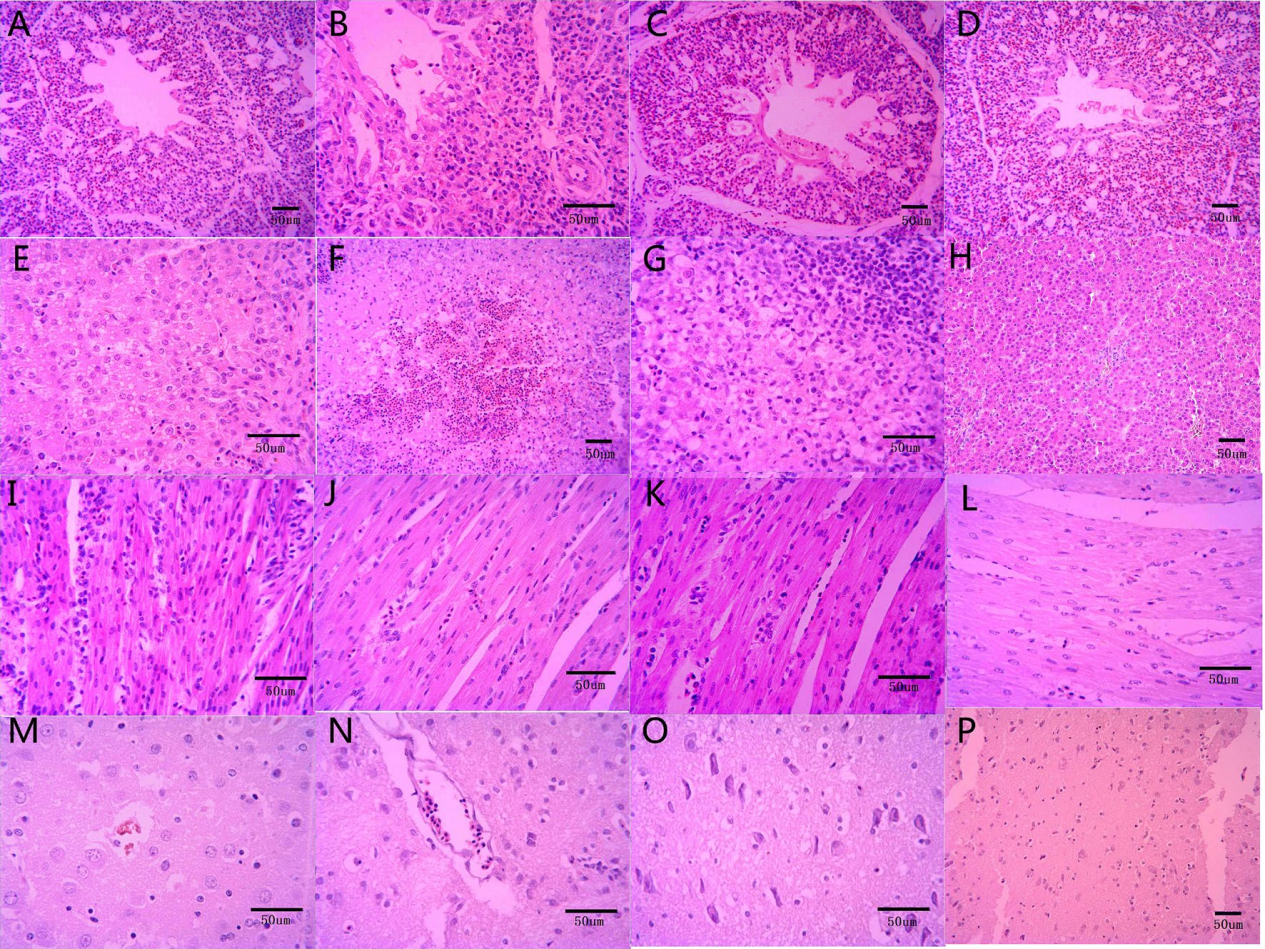
Microscopic pathological changes in lung, liver, heart and brain of chickens infected with NDRV by the s.c. route. (**A**) Lung, 3 dpi: mild interstitial pneumonia with inflammatory cell infiltration and haemorrhage; (**B**) Lung, 6 dpi: Moderate interstitial pneumonia; (**C**) Lung, 9 dpi: Mild congestion and lymphocyte infiltration; (**D**) Lung, the control group: the detection of erythrocytes in parabronchi and air capillaries; (**E**) Liver, 3 dpi: severe hepatocyte steatosis and necrosis (**F**) Liver, 6 dpi: severe hepatocyte degeneration, necrosis, inflammatory cell infiltration and focal bleeding; (**G**) Liver, 9 dpi: mild inflammatory cell infiltration; (**H**) Liver, the control group: no obvious lesions; (**I**) Heart, 3 dpi: moderate inflammation and granularity of the cytoplasm in cardiomyocytes; (**J**) Heart, 6 dpi: mild inflammatory cell infiltration; (**K**) Heart, 9 dpi: mild inflammatory cell infiltration; (**L**) Heart, the control group: no microscopic lesions; (**M**) Brain, 3 dpi: no obvious microscopic lesions; (**N**) Brain, 6 dpi: no obvious microscopic lesions; (**O**) Brain, 9 dpi: no obvious microscopic lesions; (**P**) Brain, the control group: no obvious microscopic lesions.

Microscopic lesions are summarized in Table 2. At 3 dpi, four out five (4/5) chickens showed mild interstitial pneumonia with inflammatory cell infiltration and haemorrhage (Fig. 4A). Moderate interstitial pneumonia was observed in the lungs of 4/5 infected chickens at 6 dpi (Fig. 4B). Mild congestion and lymphocyte infiltration were observed in 4/5 chickens at 9 dpi (Fig. 4C). In the control group, the detection of erythrocytes in parabronchi and air capillaries was attributed to the killing and sampling procedure. (Fig. 4D). Livers of all infected chickens at 3 dpi showed severe hepatocyte steatosis and necrosis (Fig. 4E). At 6 dpi hepatocyte degeneration, necrosis and inflammatory cell infiltration were more severe (Fig. 4F). Focal bleeding was obviously observed (Fig. 4F). At 9 dpi, inflammatory cell infiltration was mild (Fig. 4G). No lesions were found in livers of the control group (Fig. 4H).

**Table 2:** Microscopic lesions in infected chickens of two different groups at 3 dpi and 6dpi.

Organ	Experimental group			Control group		
	3 dpi	6 dpi	9 dpi	3 dpi	6 dpi	9 dpi
**Lung**						
Lesion rate	4/5^a^	4/5	4/5	0/5	0/5	0/5
Evaluation	+^b^	++	+	−	−	−
**Liver**						
Lesion rate	5/5	5/5	5/5	0/5	0/5	0/5
Evaluation	+++	+++	++	−	−	−
**Heart**						
Lesion rate	4/5	4/5	3/5	0/5	0/5	0/5
Evaluation	++	+	+	−	−	−
**Brain**						
Lesion rate	0/5	0/5	0/5	0/5	0/5	0/5
Evaluation	−	−	−	−	−	−
**Spleen**						
Lesion rate	5/5	5/5	5/5	0/5	0/5	0/5
Evaluation	+++	+++	++	−	−	−
**Duodenum**						
Lesion rate	0/5	4/5	4/5	0/5	0/5	0/5
Evaluation	−	+	+	−	−	−
**Bursae**						
Lesion rate	5/5	5/5	5/5	0/5	0/5	0/5
Evaluation	+	++	+	−	−	−
**Kidney**						
Lesion rate	4/5	4/5	4/5	0/5	0/5	0/5
Evaluation	+	++	+	−	−	−

^a^Number of chickens exhibiting microscopic lesions in the sacrificed chickens infected with NDRV.

^b^Severity of microscopic lessions: − , no microscopic lesions; +, mild microscopic lesions; ++, moderate microscopic lesions; +++, severe histological lesions.

Hearts of 4/5 infected chickens showed moderate microscopic lesions at 3 dpi and lesions began to improve at 6 dpi and 9 dpi. At 3 dpi, there was moderate inflammation and cardiomyocytes showed granularity of the cytoplasm (Fig. 4I). Mild inflammatory cell infiltration was found in 4/5 infected animals at 6 dpi (Fig. 4J) and 3/5 chickens at 9 dpi. (Fig. 4K). No microscopic lesions were observed in the hearts of control chickens (Fig. 4L). No obvious microscopic lesions of brains were observed in the infected chickens (Fig. 4M–O) or control group (Fig. 4P).

**Figure 5:**
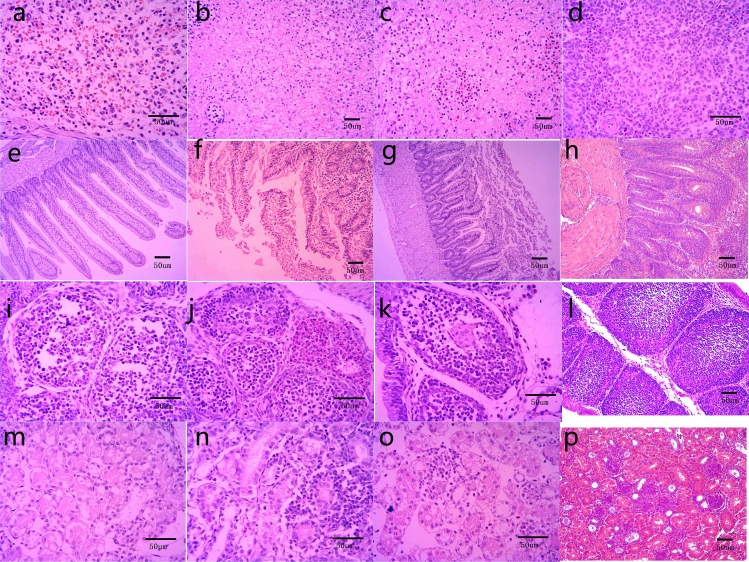
Microscopic pathological changes in spleen, duodenum, bursa and kidney of chickens infected with NDRV by the s.c. route. (**a**) Spleen, 3 dpi: severe lymphocyte depletion, haemorrhage and necrosis; (**b**) Spleen, 6 dpi: many splenic lymphocyte nuclei became pyknotic, and severe focal necrosis was observed; (**c**) Spleen, 9 dpi: moderate lymphocyte necrosis and bleeding; (**d**) Spleen, the control group: no obvious microscopic lesions; (**e**) Duodenum, 3 dpi: no obvious microscopic lesions; (**f**) Duodenum, 6 dpi: detachment of mucosal epithelium at the tip of villi; (**g**) Duodenum, 9 dpi: detachment of mucosal epithelium at the tip of villi.; (**h**) Duodenum, the control group: no obvious microscopic lesions; (**i**) Bursa, 3 dpi: lymphocyte depletion; (**j**) Bursa, 6 dpi: heterophilic granulocytes were increased and infiltrating in the cortex; (**k**) Bursa, 9 dpi: more heterophilic granulocyte infiltrating; (**l**) Bursa, the control group: no obvious microscopic lesions; (**m**) Kidney, 3 dpi: swelling and cytoplasmic granularity in the tubules; (**n**) Kidney, 6 dpi: degeneration and necrosis of tubule cells and interstitial inflammatory cell infiltration; (**o**) Kidney, 9 dpi: more heterophilic granulocytes infiltrating; (**p**) Kidney, the control group: no obvious microscopic lesions.

In the spleens all infected chickens showed severe lymphocyte depletion, haemorrhage and necrosis at 3 dpi (Fig. 5a). Also, at 6 dpi, many splenic lymphocyte nuclei became pyknotic, and severe focal necrosis was observed (Fig. 5b). Lesions were less pronounced at 9 dpi, with moderate lymphocyte necrosis and haemorrhage (Fig. 5c). No microscopic lesions of spleens were observed in the control group (Fig. 5d). Lesions were not obvious in the duodenum at 3 dpi (Fig. 5e). Duodenums of four infected chickens all showed detachment of mucosal epithelium at the tip of villi and at 6 dpi and 9 dpi. (Fig. 5f,g). No microscopic lesions of duodenums were observed in the control group (Fig. 5h).

The bursa of all infected chickens showed microscopic lesions at 3 dpi, 6 dpi and 9 dpi. Lymphocyte depletion was observed at 3 dpi (Fig. 5i). Heterophilic granulocytes were increased and infiltrating in the cortex at 6 dpi (Fig. 5j). There was more heterophilic granulocytes infiltrating at 9dpi (Fig. 5k). Microscopic lesions in bursa were not present in the control group (Fig. 5l). In the kidney, tubules showed swelling and granularity of the cytoplasm at 3 dpi in 4/5 animals (Fig. 5m). Moderate alterations were observed in 4/5 chickens at 6 dpi, including inflammatory cell infiltration (Fig. 5n). At 9 dpi granularity of the cytoplasm was mild in 4/5 animals with few inflammatory cells were observed (Fig. 5o). No microscopic lesions of kidney were observed in the control group (Fig. 5p).

### Immunohistochemical examinations

**Figure 6:**
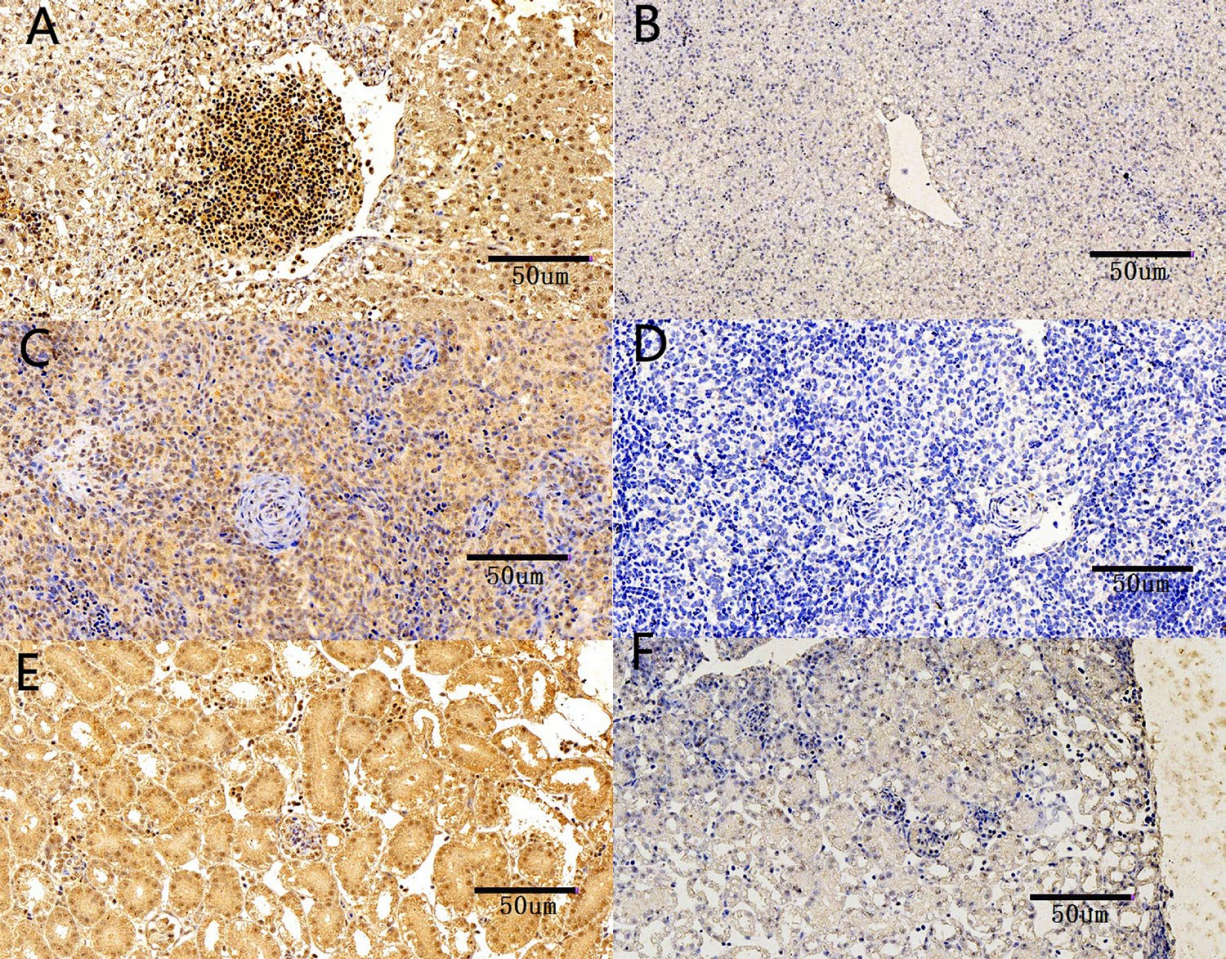
Immunohistochemical examination of NDRV antigen in the liver, spleen and kidney at 6 dpi. (**A**) The positive immunohistochemical staining signals were distributed in liver. (**B**) The control group: no obvious positive signals were in liver. But slight background staining was observed. (**C**) Positive signals were distributed in spleen. (**D**) The control group: no obvious positive signals were in spleen. (**E**) Strong positive signals were distributed in the renal tubular epithelial cells. (**F**) The control group: no obvious positive signals were in kidney. But slight background staining was observed.

Viral antigens in the tissue sections were stained brown by the immunohistochemical examinations at 6 dpi. In the liver, positive immunohistochemical staining signals were mainly distributed in the liver cytoplasm and nuclei. The nuclei of red blood cells within the hepatic sinus gap were also positively stained (Fig. 6A). In the brain, positive staining signals were widely distributed in the cytoplasm of the neurons, and part of the nucleolus was positively stained (Fig. 6C). In the spleen, positive signals were widely distributed in the red pulp. The concrete localization of positive signals was in the nucleus of the splenocytes (Fig. 6E). In the kidney, strong positive signals were mainly focused on the renal tubular epithelial cell cytoplasm and nuclei. Part of the nuclei was strongly positively stained. The glomerular podocyte cytoplasm also presented positive reactions (Fig. 6G). No obvious positive signals were observed in the control groups (Fig. 6B,D,F,H). The background staining was observed in the control of liver, brain and kidney, but it was very slight (Fig. 6B,D,H).

### Viral loads in different tissues

**Figure 7:**
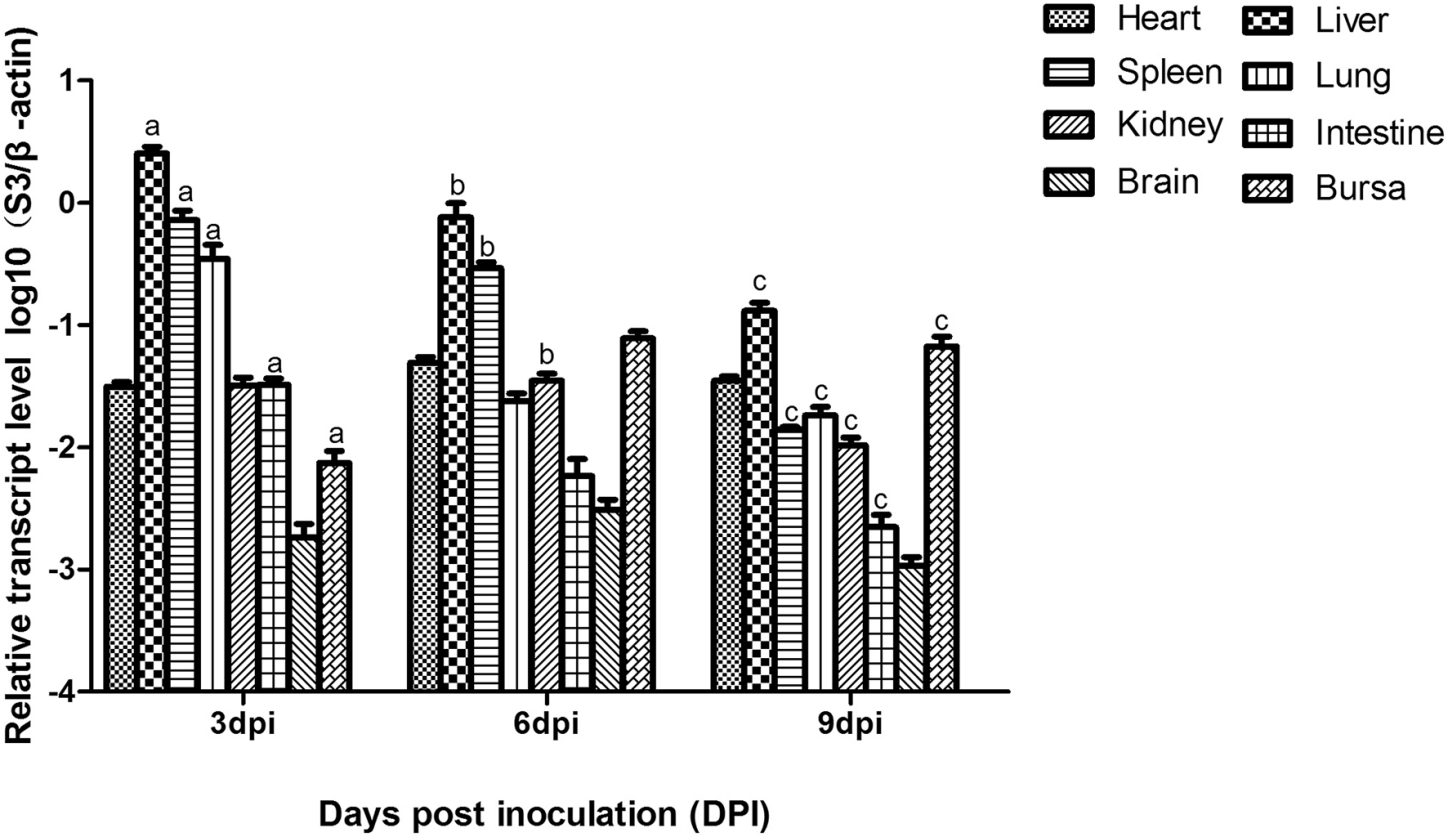
Viral loads in different tissues of chickens infected with NDRV by the s.c. route. The relative expression of viral RNA in tissues was determined by SYBR Green I real-time PCR assay (S3 gene as a target gene and chicken β-actin gene as a reference gene). Each sample was tested in triplicate. Error bars were expressed as standard deviation of the means (n = 3). Data are expressed as the mean log10 (E/β-actin) ± SD. Different lowercase letters over the bars showed statistically significant differences (P < 0.05) between different days after infection. The data were calculated by the one-way analysis of variance with Tukey’s post-test. (a) 3 dpi vs 6 dpi; (b) 6 dpi vs 9 dpi; (c) 3 dpi vs 9 dpi.

Viral loads were detected in the heart, liver, spleen, lung, kidney, brain, intestines and bursae of infected chickens by a SYBR Premix EX Taq assay. It can be seen in Fig. 7 that the viral loads in the liver, spleen, lung, kidney and intestines reached the peak at 3 dpi. In particular, the viral loads in the liver, spleen and lung were much higher than those in the other tissues. The level of viral RNA in different tissues began to decline from 6 dpi, but the viral loads in the liver and spleen always remained at a higher level before 6 dpi, and this result was consistent with the mortality of chickens infected with NDRV. The level of viral RNA in the bursae reached a peak at 6 dpi and maintained a higher expression level. Viral loads of the brain always remained at a lower level. No viral RNA was detected in the control group.

### Detection of the serum neutralizing antibody

**Figure 8:**
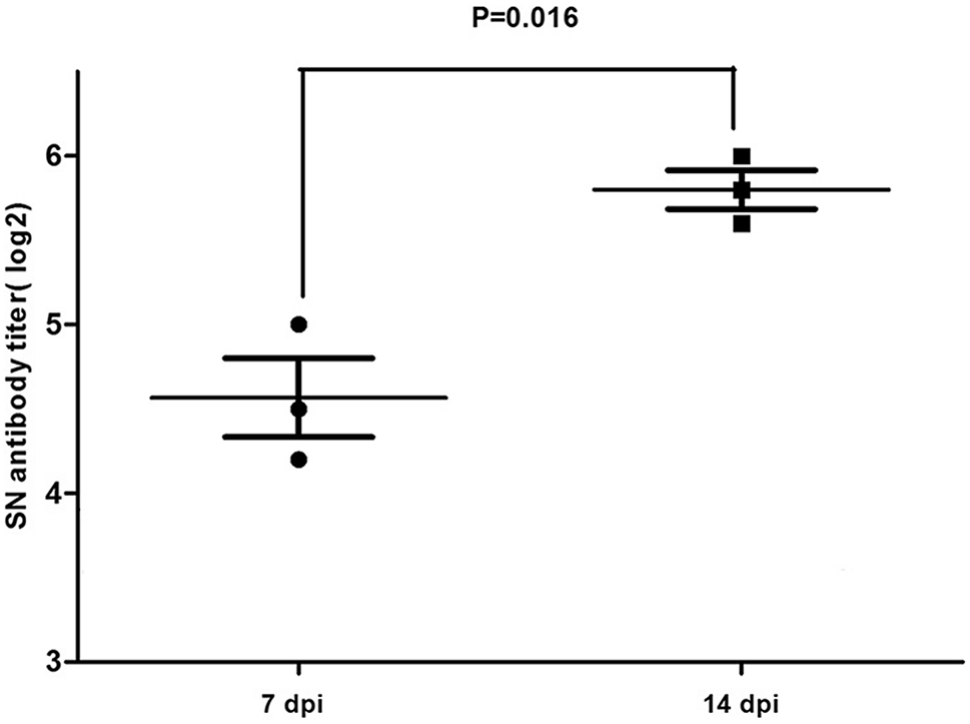
Serum neutralizing antibody against NDRV was detected. Each sample was tested in triplicate. Serum neutralizing antibody titers were expressed as the reciprocal of the log2 of the highest dilution serum dilution that inhibited 50% CEF death. The negative antibody titer was zero. Data are expressed as mean ± SD (n = 3). Significant differences were determined by the two-tailed Student’ s unpaired t test.

As seen in Fig. 8, chickens infected with NDRV induced neutralizing antibodies at 7 dpi (SN antibody titre > 10). From 7 dpi, the neutralizing antibody titres were continuously ascending, and the level at 14 dpi reached a much higher level. No positive neutralizing antibody titres were detected in chickens in the control group (SN antibody titre < 10).

## Discussion

NDRV infection has become a common disease in the Chinese duck industry. Although its mortality rate has not been high, the disease can cause spleen necrosis and immune suppression, which can develop into a serious secondary infection and growth retardation in ducks. More importantly, NDRV infection is a disease that can spread horizontally, as well as vertically, and the offspring of infected breed ducks can easily transmit the virus and cause disease^17^. Therefore, NDRV infection is much more difficult to prevent and control in practical production. NDRV can infect different types of ducks and cause diseases, but research on the pathogenicity of NDRV to chickens has not been reported until now. This study was focused on the clinical symptoms, pathological changes, viral RNA expression, and serum antibodies of infected chickens to further evaluate the pathogenicity of NDRV to chickens.

It was reported that NDRV can infect 10-day-old chicken embryos by allantoic cavity inoculation. Chickens were delayed in hatching and had obvious necroses in the liver and spleen^6,16^. In this study, 3-day-old chickens infected with NDRV subcutaneously exhibited body weight loss, introflexion of claws, performing of splits, death, etc. The most typical gross lesions mainly included swelling, brittleness, and yellowish-white focal necroses in the liver and spleen. These experimental data were consistent with the symptoms and lesions of ducks infected with NDRV^6,16,17^. In previous studies, lymphocyte depletion could be observed in most of the tissues of ducks infected with NDRV^14,17,18^. In this study, similar lesions were also found in different chicken organs. For example, inflammatory cell infiltration was severe in the liver, while lymphocyte depletion was obvious and typical in the spleen and bursa. The spleen and bursa are very important immune organs in poultry, pathological damage to which can lead to the immunosuppression of the body. In particular, the bursa plays an important role in inducing B lymphocytes to differentiate and mature, so lymphocyte depletion could lead to immune dysfunction.

In addition to the liver, spleen and bursae, other tissues also demonstrated varying degrees of pathological changes, such as lymphocyte infiltration with congestion in the lung, cytoplasmic granularity in the myocardium of the heart with lymphocyte infiltration, cytoplasmic granularity in renal tubules, etc. Most studies of the pathogenicity of NDRV mainly focused on the immune organs of poultry, and other organs were rarely involved^7,12,14,15,18,19^. However, in this study most of the organs of the chickens were observed to develop severe lesions, which was in agreement with the pathogenicity study of NDRV to ducks^17^. It was reported that the lesions of brain were slight in ducks^17^. In this study no lesions were observed in the chicken brains. It also means that tissue tropism of MDRV to brain is very slight and there were differences between chickens and ducks.

In this study, the viral RNA of most tissues reached the highest level at 3 dpi, and the level of viral RNA expression began to decline from 6 dpi. The data suggested that the developing speed of the disease was much faster, consistent with the deaths among diseased chickens being mainly focused in the period of before 6 dpi. The results were also consistent with the dynamic changes in viral loads in duck tissues^17^. In this study, pathological changes in the spleen and liver were much more serious at 6 dpi, and they were closely related to the level of the viral loads being maintained for a longer time in these tissues. The viral loads of the liver and spleen were much higher than those in other tissues and were maintained for a longer period, consistent with the severe and obvious lesions in the two organs. Compared to other tissues, the viral loads of the bursae reached the peak level at 6 dpi and always maintained a higher level until 9 dpi. These data indicated that the liver, spleen and bursae of chickens could be the target organs of NDRV.

The viral loads of the brain always maintained at a lower level which consistent with the pathological characteristics of the brain.

In this study, the chickens could produce neutralizing antibodies to NDRV at 7 dpi. This finding indicated that NDRV could infect chickens subcutaneously and induce neutralizing antibodies.

This study indicates that chickens can be infected subcutaneously with a virulent NDRV strain that can cause disease or even death. However in practical poultry production chickens and ducks were rarely mixed, so chickens were not easy to be exposed to the virus. Even if chickens were exposed to NDRV, it was very difficult for chickens to infect a high-dose virus a time in practical poultry production. Therefore, there have been no cases infected with NDRV in chicken production. But if the frequent exposure to NDRV always remains, which could lead to virus variation and the virus will be probably susceptible to chickens. And that reovirus is a multi-segment RNA virus^1^, chicken-derived reovirus and duck-derived reovirus are more likely to exchange and recombine. It suggests that the virus may infect chickens naturally in practical poultry production in the future. Therefore in the actual production, chickens and ducks should avoid being mixed to breed and thus chickens will avoid being exposed to the duck-derived viruses, such as NDRV. And chickens will also avoid adapting to the virus due to long-time exposure of NDRV. Although so far chickens have not been naturally infected with the NDRV strain, there is still a great risk of infection. This study provided some experimental data for further prevention and control of infection by NDRV in poultry.

## Materials and methods

### Animals and virus

Three-day-old SPF chickens and 9-day-old SPF duck embryos were purchased from Shandong Hao Tai Experimental Animal Breeding Company Limited. The novel duck reovirus (NDRV) strain was isolated from one Cherry Valley duck farm in Shandong Province in 2012. The virus was named SD-12 (Accession number: KJ879930). It was inoculated into the allantoic cavities of 9-day-old SPF duck embryos for subculture. After three passages, the virus was used for the challenge in this study. The virus titre was 10^5.00^ ELD_50_/0.1 mL (50% lethal dose for embryos) for the stock, determined according to the method of Reed and Muench^20^. The virus was isolated by our team members. PCR detection showed that there were not duck enteritis virus, duck hepatitis A virus, duck tembusu virus, duck parvovirus and other common duck virus in the allantoic fluid.

### Animal experiments

Eighty 3-day-old SPF chickens were raised in negative pressure isolators and randomly divided into two groups, with 40 chickens in each group. The experimental group was inoculated with 100 μL of allantoic fluid (10^5.00^ ELD_50_/0.1 mL) of NDRV by the s.c. route. The other group, as the controls, was inoculated with 100 μL of PBS. The virus dose was determined by a pre-test. Clinical symptoms of the two groups were observed every day. Ten chickens were randomly selected from each group and weighed every three days for 14 days. Blood of the infected chickens was collected and analysed every day. At 3, 6, 9 dpi, five chickens from each group were euthanized by carbon dioxide, and their tissues (heart, liver, spleen, lung, kidneys, brain and intestine) were collected. One part of the tissue sample was fixed in 10% neutral buffered formalin solution for histological examination. The other part of the tissue sample was stored at − 80 °C until use for RNA extraction. The test animal bodies, embryo bodies and the used test materials were disposed harmlessly. The whole animal experiments were conducted in the Biosafety Level 2 laboratory.

### RNA extraction and reverse transcription

Total RNA of different frozen tissue samples was extracted using a Total RNA Extraction Kit (Solarbio, Beijing, China). The RNA concentration of tissue samples was measured by an automatic nucleic acid analyser (Eppendorf, Germany). Complementary DNA (cDNA) of 1000 ng of RNA was synthesized with a PrimeScript RT reagent kit with gDNA eRaser (TaKaRa, DaLian, China). The volume of the reverse transcription reaction system was 20 µL.

### Viral load detection

The viral loads of different tissue samples were detected by a SYBR Premix EX Taq (Perfect Real-time) assay^11^. NDRV S3 gene and duck β-actin gene are respectively as a target gene and a reference gene. Two pairs of primers were designed according to S3 gene sequence (GenBank No. KJ879932) and β-actin gene sequence (GenBank No. NM_205518.1). The S3 gene primers were as follows, F: 5′-ATGTCGCTGTCACGGGTAA-3′ and R: 5′-TGGTAGGAACCACGCTCAA-3′. The size of amplified fragment was 196 bp. The β-actin gene primers were as follows, F: GTGCTGTGTTCCCATCTATC and R: TTTGCTCTGGGCTTCATC. The size of amplified fragment was 101 bp. The amplification system, which was 25 μL in volume, contained the following components: 1.0 μL of forward primer (10 μmol L^−1^), 1.0 μL of reverse primer (10 μmol L^−1^), 1 μL of cDNA template, 12.5 of μL 2 × SYBR Premix Ex Taq (TaKaRa, DaLian, China) and 9.5 of μL sterilized deionized water. The PCR thermal cycles comprised the following steps: 95 °C for 45 s, 40 cycles of 94 °C for 10 s, 56 °C for 10 s and 72 °C for 15 s. Each sample was detected in triplicate.

### Histopathology and immunohistochemistry examinations

Tissue samples were fixed in 10% neutral buffered formalin solution for 72 h, dehydrated, and embedded in paraffin wax. Sections that were 4 µm thick were cut. One part of the sections was stained with haematoxylin and eosin (H&E) following the standard histopathological protocols, and the pathologic results were observed under a microscope.

The other part of the sections at 6 dpi was deparaffinized with xylene and hydrated with different grades of alcohol liquid (100–75%) for the immunohistochemical examination. 0.01 M sodium citrate buffer solution (pH6.0) was heated to 95 °C and then sections were immersed for 10 ~ 15 min to retrieve the antigen. After blocking with 5% goat serum albumin buffers for 1 h, the sections were incubated with rabbit sera against NDRV overnight at 4 °C. After three washes with PBS, the sections were conjugated with a diluted mouse anti-rabbit HRP-conjugated polyclonal serum for 1 h at 37 °C. Diaminobenzidine could be used as the substrate chromogen. After counterstaining with haematoxylin, the sections were sealed with neutral gum and observed with the microscope. Rabbit sera against NDRV were prepared by our own laboratory. Purified and concentrated NDRV was used as the immunological antigen. The acquired antisera on rabbit anti-NDRV didn't react with the main chicken viruses such as newcastle disease virus, avian influenza virus, infectious bronchitis virus, infectious laryngotracheitis virus, infectious bursal disease virus, avian leukosis virus and chicken reovirus by a serum neutralization test. The antisera also didn't react with the main duck viruses such as duck enteritis virus, duck hepatitis A virus, duck tembusu virus, duck parvovirus and other common duck virus by a serum neutralization test. It was diluted by 1:200 and used as the primary antibody of the immunohistochemistry examinations.

### Analysis of serum antibodies against NDRV

Serum neutralization testing (SNT) was used to detect the serum antibody titres against NDRV. Serum samples of three chickens were randomly collected respectively at 0 dpi, 7 dpi and 14 dpi, respectively. Before starting the tests, complements in serum samples had to be inactivated at 56℃ for 30 min. SNT was performed with duck embryo fibroblasts (DEF) as previously described^12,17^. Serum neutralizing antibody titres were expressed as the reciprocal of the log2 of the highest serum dilution that inhibited 50% DEF death and was calculated by the method of Reed and Muench^20^. Each sample was performed in triplicate.

### Statistical analysis

The experimental test data are expressed as the means ± standard deviations. Serum antibody titres and body weight data were analysed with Student’s two-tailed unpaired t-test. Viral loads in different tissues were evaluated by one-way analysis of variance (ANOVA) with Tukey’s post-test. Statistical significance was represented by P < 0.05 and P < 0.01.

### Ethics statement

The animal experiments were approved by the Committee on the Ethics of Animal of Institute of Poultry Science, Shandong Academy of Agricultural Sciences (permit number: 2019005), according to the guidelines of the Review of Welfare and Ethics of Laboratory Animals authorized by the Shandong Municipality Administration Office of Laboratory Animals. The animal Experiments were conducted in the Biosafety Level 2 laboratory in Shandong Academy of Agricultural Sciences, in compliance with the ARRIVE guidelines. All applicable international, national, and/or institutional guidelines for the care and use of animals were followed. The Ethical approval and ARRIVE guidelines are as follows.

## References

[1] Javier B, Jose MC (2007). Avian reovirus: Structure and biology. Virus Res..

[2] Olson NO, Kerr KM (1966). Some characteristics of an avian arthritis viral agent. Avian Dis..

[3] Chen S, Chen S, Cheng X, Jiang B, Lin F, Wang S (2011). The comparison of serologic relativity and CPE types of 3 avian reovirus strains induced different disease. Acta V. Zootech. Sin..

[4] Olson NO, Weiss R (1972). Similarity between arthritis virus and Fahey-Crawley virus. Avian Dis..

[5] Yun T, Yu B, Ni Z, Ye W, Chen L, Hua J (2013). Isolation and genomic characterization of a classical Muscovy duck reovirus isolated in Zhejiang, China. Infect. Genet. Evol..

[6] Zheng X, Wang D, Ning K, Liang T, Wang M, Jiang M (2016). A duck reovirus variant with a unique deletion in the sigma C gene exhibiting high pathogenicity in Pekin ducklings. Virus Res..

[7] Bi Z, Zhu Y, Chen Z, Li C, Wang Y, Wang G (2016). Induction of a robust immunity response against novel duck reovirus in ducklings using a subunit vaccine of sigma C protein. Sci. Rep..

[8] Chen S, Chen S, Lin F, Wang S, Jiang B, Cheng X (2012). The isolation and identification of novel duck reovirus. Chin. J. Virol..

[9] Liu Q, Zhang G, Huang Y, Ren G, Chen L, Gao J (2011). Isolation and characterization of a reovirus causing spleen necrosis in Pekin ducklings. Vet. Microbiol..

[10] Chen Z, Zhu Y, Li C, Liu G (2012). Outbreak-associated novel duck reovirus, China, 2011. Emerg. Infect. Dis..

[11] Pan L, Ma X, Huang Z, Li G, Tang L (2020). Study on anti-novel duck reovirus effect of chlorogenic acid in vitro. J. Agric. Biotechnol..

[12] Li S, Gu C, Bai J, Zhang H, Zhang W, Cheng G (2010). Observation of immunological damage of duckings infected by duck reovirus. Chin. Agric. Sci..

[13] Li Z, Cai Y, Liang G, Ei-Ashram S, Mei M, Huang W (2018). Detection of novel duck reovirus (NDRV) using visual reverse transcription loop-mediated isothermal amplification (RT-LAMP). Sci. Rep..

[14] Zhang Y, Diao Y, Gao M, Ge P, Sun X, Lu A (2015). Pathogenicity research of ducklings infected by duck reovirus. Chin. Acad. Vet..

[15] Wang H, Gao B, Chen H, Diao Y, Tang Y (2019). Isolation and characterization of a variant duck orthoreovirus causing spleen necrosis in Peking ducks, China. Transbound Emerg. Dis..

[16] Liu X, Liu T, Liu B, Cheng G, Gu C, Zhang W (2016). The pathogenicity of duck reovirus on SPF chicken embryo. Sci. Agric. Sin..

[17] Li N, Hong T, Wang Y, Wang Y, Yu K, Cai Y (2016). The pathogenicity of novel duck reovirus in Cherry Valley ducks. Vet. Microbiol..

[18] Cao Y, Sun M, Wang J, Hu X, He W, Su J (2019). Phenotypic and genetic characterization of an emerging reovius from Pekin ducks in China. Sci Rep..

[19] Zhao H, Li S, Gu C, Zhang W, Chen G, Liu X (2015). Study on pathology and distribution of duck reovirus in naturally infected ducks. Acta Vet. Zootech. Sin..

[20] Reed LJ, Muench H (1938). A simple method of estimating fifty per cent endpoints. Am. J. Epidemiol..

